# Kinematic and biomechanical responses of the spine to distraction surgery in children with early onset scoliosis: A 3-D finite element analysis

**DOI:** 10.3389/fbioe.2022.933341

**Published:** 2022-07-15

**Authors:** Baoqing Pei, Da Lu, Xueqing Wu, Yangyang Xu, Chenghao Ma, Shuqin Wu

**Affiliations:** ^1^ Beijing key laboratory for design and evaluation technology of advanced implantable and interventional medical devices, Beijing Advanced Innovation Center for Biomedical Engineering, School of Biological Science and Medical Engineering, Beihang University, Beijing, China; ^2^ School of Big Data and Information, Shanxi College of Technology, Shanxi, China

**Keywords:** spinal distraction surgery, bilateral fixation, kinematic and biomechanical response, law of diminishing return, traditional growth rod

## Abstract

Periodical and consecutive distraction is an effective treatment for severe early onset scoliosis (EOS), which enables the spinal coronal and sagittal plane deformity correction. However, the rate of rod fractures and postoperative complications was still high mainly related to the distraction process. Previous studies have primarily investigated the maximum safe distraction force without a rod broken, neglecting the spinal re-imbalance and distraction energy consumption, which is equally vital to evaluate the operative value. This study aimed to reveal the kinematic and biomechanical responses occurring after spinal distraction surgery, which were affected by traditional bilateral fixation. The spinal models (C6-S1) before four distractions were reconstructed based on CT images and the growing rods were applied with the upward displacement load of 0–25 mm at an interval of 5 mm. Relationships between the distraction distance, the distraction force and the thoracic and lumbar Cobb angle were revealed, and the spinal displacement and rotation in three-dimensional directions were measured. The spinal overall imbalance would also happen during the distraction process even under the safe force, which was characterized by unexpected cervical lordosis and lateral displacement. Additionally, the law of diminishing return has been confirmed by comparing the distraction energy consumption in different distraction distances, which suggests that more attention paid to the spinal kinematic and biomechanical changes is better than to the distraction force. Notably, the selection of fixed segments significantly impacts the distraction force at the same distraction distance. Accordingly, some results could provide a better understanding of spinal distraction surgery.

## Introduction

EOS is a progressive spinal deformity that occurs in children before the age of 10 years. Unilateral or bilateral posterior fixation such as the growing rod technique was used to limit the progression of scoliosis without stunting the spinal growth (Stokes et al., 1996; Akbarnia et al., 2005, Akbarnia et al., 2008; Thompson et al., 2007; Villemure and Stokes, 2009; Elsebai et al., 2011). It requires repeat distraction every 6 or more months via open surgery under general anesthesia (Akbarnia et al., 2005, 2008; Sankar et al., 2011). However, the situations such as postoperative complications, rod fractures and second spinal imbalance are still existing. For example, more than 50% of patients treated with growing rods had at least one complication at some point after surgery (Bess et al., 2010). Similarly, rod fractures occured in 15% of patients (Thompson et al., 2005; Bess et al., 2010; Yang et al., 2011), which were perhaps accompanied by screw loosening (Li et al., 2010). Higher or lower force, suboptimal distraction could also lead to poor sagittal contours in juvenile patients (Akbarnia et al., 2005). These conditions are related to the choice of distraction force and distraction frequency, which depends on the patient’s growth.

The recent studies contributed to a better understanding of the relationship between distraction force and distraction frequency. A shorter distraction episode can effectively reduce the stress on the rods while patients must undergo more surgical damage (Agarwal, et al., 2014a; Agarwal, et al., 2014b, Agarwal, et al., 2015, Agarwal, et al., 2017; Agarwal, et al., 2017a). Meantime, frequent distractions are more gentle on the soft tissues and may avoid progressive stiffness of auto fusion of the spinal segments (Cheung et al., 2016). On the other hand, a greater distraction force resulted in a significant therapeutic effect in a short time and a reduction of surgical damage, which also meant an increased risk of rod fracture (Agarwal et al., 2017b). However, little attention has been paid to the overall spinal balance and local biomechanical environment changes after distraction surgery.

Agarwal et al. (2017) simulated 6 months of spinal growth under various distraction forces and found that the optimal force existed in all types of scoliotic curves. Optimization of the biomechanical environment could reduce the complications associated with growing rods. The force threshold that the rod can withstand may not be applicable to the bones. They paid more attention to the stress on rods that neglected the biomechanical changes on the spine or vertebrae. Nevertheless, Justin V.C. Lemans et al. (Jvcl et al., 2021) investigated the destructive force by stretching the spine *in vitro* and found the maximum force threshold was 800–1200N (age >5). They did not focus on the issues of spinal coronal or sagittal imbalance. Nail rod system has more stable characteristics than spine. So, it is more effective to focus on the spinal response after distraction surgery.

No prior study has ever systematically investigated the local or global kinematic and biomechanical response after spinal distraction. Therefore, the current study aimed to reveal the spinal biomechanical changes in the cases of growing rods distracting at different distances. In particular, the distraction force, reduction of Cobb angle, spinal movement and rotation in three-dimension, coronal and sagittal balance and intervertebral disc (IVD) stress parameters were measured. The interplay between them is also interpreted to aid in developing and optimizing this technology and its contemporary counterparts.

## Materials and methods

### Subjects

The research was approved by the Science and Ethics Committee of the School of Biological Science and Medical Engineering at Beihang University (protocol code: BM20220087).

**FIGURE 1:**
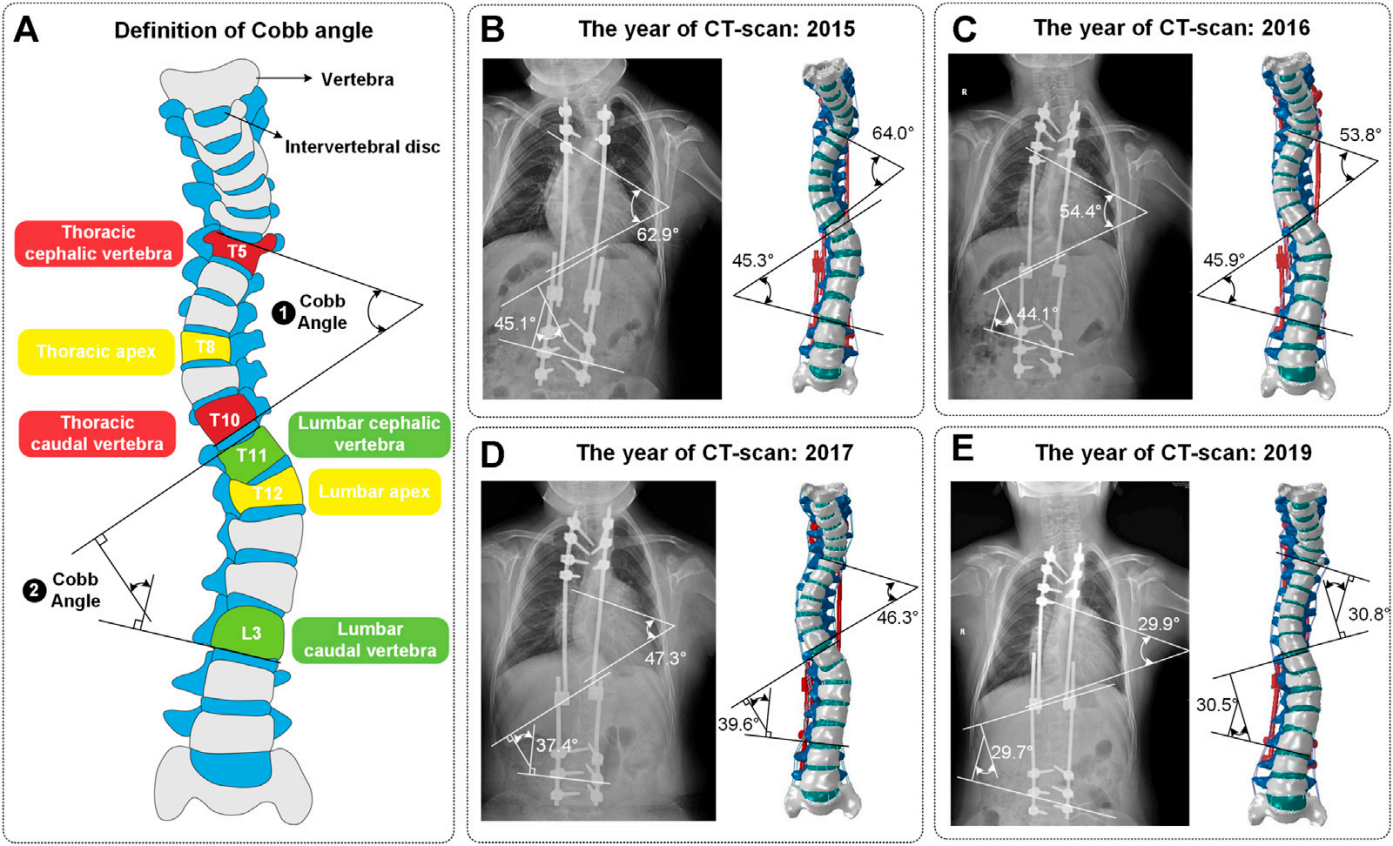
**(A)** Definition of Cobb angle. Cobb angle is the angle of intersection between the vertical of the superior edge of the cephalic vertebra and the vertical of the inferior edge of the caudal vertebra. **(B–E)** The four surgeries included one growing rod implantation and three spinal distractions. These four models were named Model_1, Model_2, Model_3 and Model_4. The Cobb angle of models conformed to the ones of CT images (mean error: 1.08°).

The patient (8 years, 115 cm, 30 kg) underwent the first growing rod implantation in 2015 due to excessive lordosis of the cervicothoracic junction. Three more surgeries for spine distraction followed in 2016, 2017, and 2019 (Figure 1). This patient did not have any other known musculoskeletal disorders. The preoperative X-ray displays a thoracic curve Cobb angle of 62.9°/54.4°/47.3°/29.9° (thoracic apex: T8; thoracic cephalic vertebra: T5; thoracic caudal vertebra: T10) and a lumbar curve Cobb angle of 45.1°/44.1°/37.4°/29.7° (lumbar apex: T12; lumbar cephalic vertebra: T11; lumbar caudal vertebra: L3), which were captured at an interval of 1 mm and a resolution of 512 × 512 px using a CT scanner (model: SOMATAM Definition Edge) from Berlin and Munich, Germany. The CT scanner has a maximum scanning speed of 230 mm/s1, a spatial resolution of 0.3 mm and a single source.

### Creating the base model

**FIGURE 2:**
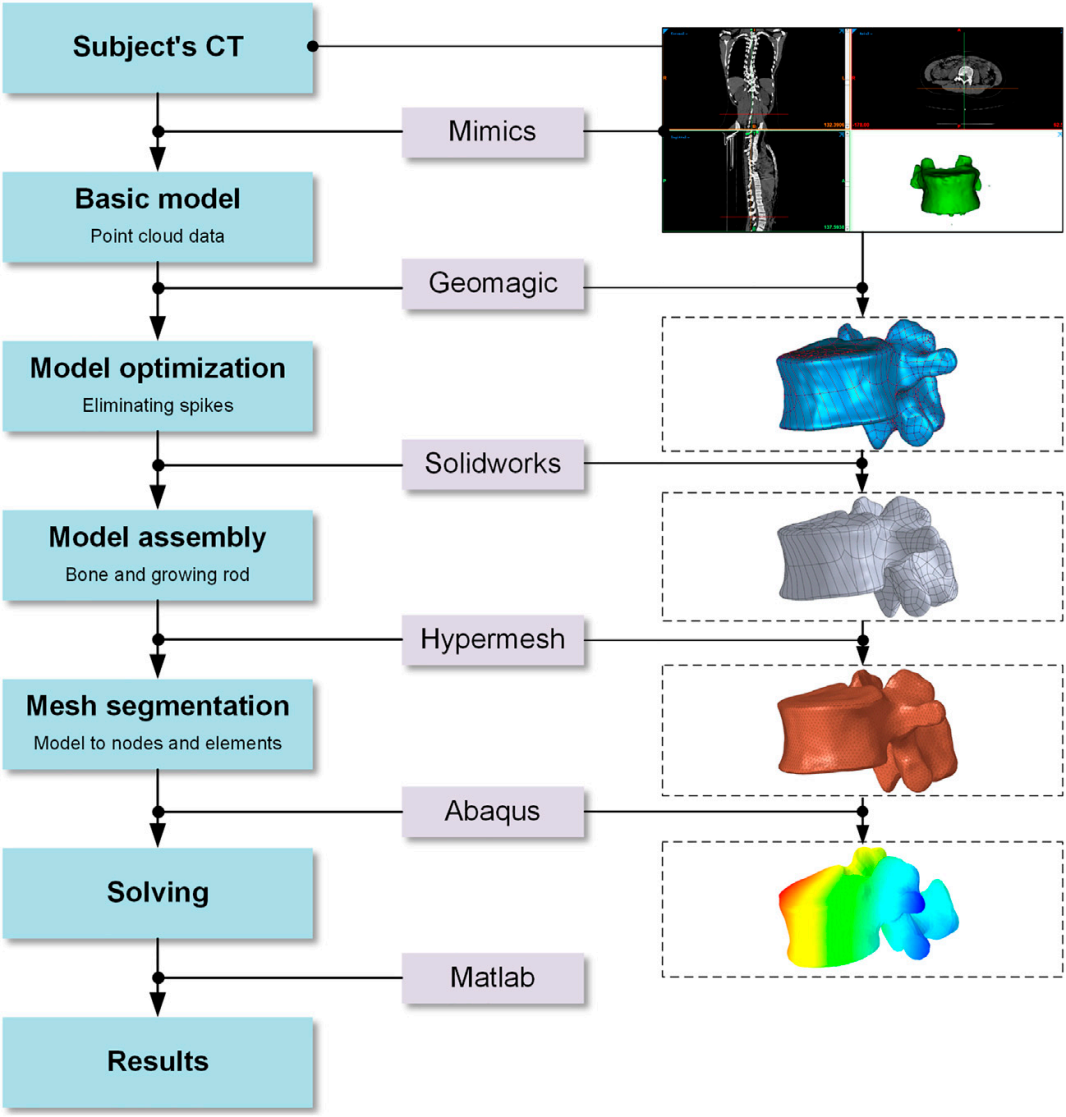
Modeling process in this study.

**FIGURE 3:**
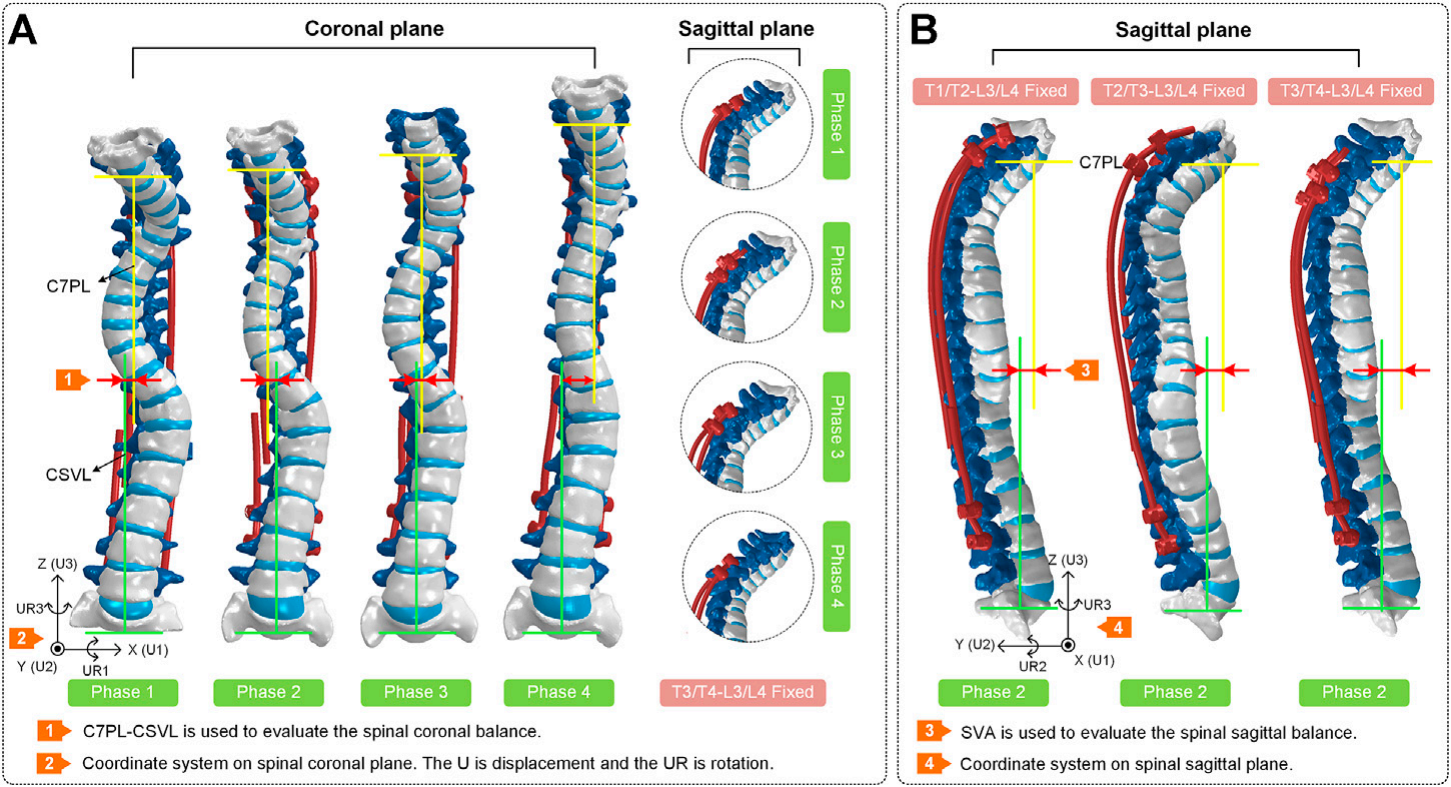
**(A)** Coronal view and sagittal view of the spines at four phases. The T3/T4-L3/L4 segments of each spine are fixed. The coronal balance parameters (C7PL-CSVL) of four models are shown to evaluate the surgical effects. **(B)** Sagittal view of spines in surgical phase 2. The T1/T2-L3/L4, T2/T3-L3/L4 and T3/T4-L3/L4 segments of each spine are fixed separately. The sagittal balance parameters (SVA) of three models are shown to evaluate the surgical effects.

The modeling process in this study has been shown in Figure 2. The initial and rough models including C6-S1 vertebrae, screws and growing rods were reconstructed based on CT images and MIMICS (version: 17.0; company: Materialise; location: Europe Belgium). After getting point clouds from MIMICS, the operations of further smoothing and surface patches divided were carried on through Geomagic Studio (version: 2013; company: Geomagic; location: Triangle, NC, United States). Then, individual CAD models were imported into a whole in SolidWorks (version: 2019; company: Dassault Systemes SE; location: Paris, France). It’s worth noting that IVDs were built by lofting the superior and inferior vertebra surface rather than CT data since IVDs contained less bony material to image on CT. In the fourth step, the rest operations such as mesh division, material properties assignment and ligaments established were finished in Hypermesh (version: 14.0; company: Altair; location: Troy, Michigan, United States). In the fifth step, these assembled models were imported into ABAQUS (version: 2016; company: Dassault SIMULIAL; location: Providence, Rhode Island, United States) for simulation. Finally, the data post-processing was carried out in MATLAB (version: 2018a; company: MathWorks; location: Natick, Massachusetts, United States). The coronal view and sagittal view of reconstructed models with different growth phases and fixed segments was shown in Figure 3.

### Adopted mesh and material properties

Bony structures and soft tissues were discretized to tetrahedral (C3D4) element type and hexahedral (C3D8) element type. For the same element size, hexahedral discretization will produce more element nodes than tetrahedral discretization which lead to the time-consuming phenomenon. This phenomenon can be avoided by discretizing some trivial structures using tetrahedral elements. This division method also achieved high accuracy and reliability. And the ligament tissues were modeled with three-dimensional truss elements and discretized in a one-dimensional line grid. These ligament tissues included the anterior longitudinal ligament (ALL), posterior longitudinal ligament (PLL), interosseous transverse ligament (ITL), capsular ligament (CL), interspinous ligament (ISL), supraspinous ligament (SSL), and ligament flavum (LF). Similarly, the IVDs were divided into fibrous annulus (AF), nucleus pulposus (NP) and cartilage endplate. Then, each material property is shown in Table 1.

**TABLE 1:** Material properties in the present finite element models.

Element construction	Element type	Elasticity modulus/MPa	Poisson ratio	Cross-sectional area/mm^2^	Scale factors	References
Cortical bone	Hexahedron	1.344e4	0.30	—	0.805(b)	(Currey, 2004; Berteau et al., 2014)
Cancellous bone	Quadrilateral	2.41e2	0.30	—	0.805(b)	Kopperdahl and Keaveny, (1998)
Posterior	Hexahedron	3.5e3	0.25	—	(a)	Rohlmann et al. (2009)
Endplate cartilage	Quadrilateral	2.38e1	0.40	—	(a)	Kurutz and Oroszváry, (2010)
Nucleus pulposus	Hexahedron	1.0e0	0.49	—	(a)	
Annulus fibrosus	Hexahedron	4.2e0	0.45	—	0.782(b)	
ALL	Three-dimensional truss	7.8e0	0.12	63.7	0.893(b)	Kurutz and Oroszváry, (2010)
PLL		1.0e1	0.11	20.0		
ITL		1.0e1	0.18	1.80		
CL		7.5e0	0.25	30.0		
ISL		8.0e0	0.14	30.0		
SSL		1.0e1	0.20	40.0		
LF		1.5e1	0.062	40.0		
Growing rod	Hexahedron	1.1e5	0.30	—	(a)	(Aakash et al., 2015)

(a) Indicates that the material parameters were the same as the values of an adult. (b) The scale factors were used to scale adult material parameters to child ones (Yoganandan et al., 2000).

Bony structures were assumed as linear elastic materials, without considering plastic deformation. Although there is overwhelming evidence that younger immature bone can undergo more plastic deformation prior to fracture than mature bone (Berteau et al., 2015; Szabo and Rimnac, 2022), the yield stress (about 100 MPa reported in (Currey and Pond, 1989) is not likely to be achieved in clinical surgery.

### Constraint setting and validation

**FIGURE 4:**
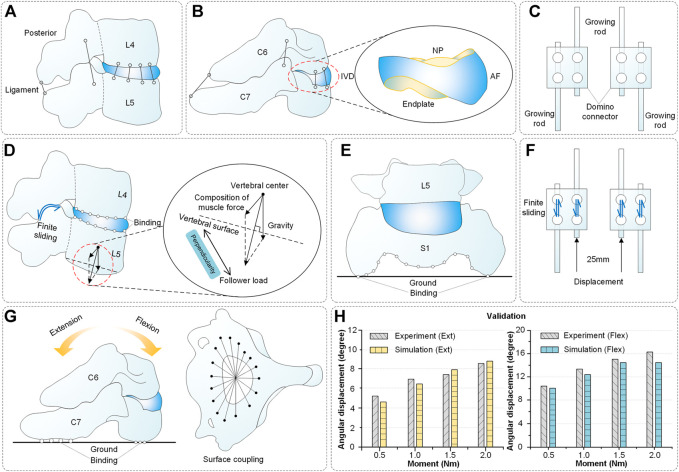
Spinal composition, constraint setting and validation. **(A-B)** Components of spinal reconstruction. **(C)** Structure of growing rods and domino connectors. **(D)** Spinal constraint setting (L4-L5 segments as an example) includes finite sliding (facet joints), binding (vertebra and IVD) and follower load (vertebrae). **(E)** Spinal constraint setting: binding (S1 segment and ground). **(F)** Constraint setting: finite sliding (growing rod and domino connector), displacement load (growing rod, 0–25 mm). **(G)** Spinal constraint setting for validation. The superior surface of C6 was coupled and applied a pure torque. The inferior surface of C7 was bound with the ground. **(H)** Validation results of the model.

Many individual components were not combined into a finite element entity. Thus, an illustration of the spinal constraint setting is shown in Figure 4. A complete functional segment unit (FSU) consists of vertebrae, IVD, ligaments and a growing rod system. Among them, the IVD includes nucleus pulposus (NP), annulus fibrosus (AF) and endplates, the growing rod system includes the growing rods, domino connectors and screws (Figures 4A–C).

Additionally, these operations were performed: the vertebrae were connected by ligament, the vertebrae and IVDs were connected by binding, the S1 segment and ground were connected by binding, the facet joints were connected by finite sliding (friction coefficient = 0.01 (Cai et al., 2019)) and the growing rod and domino connector connected by sliding too (Figures 4D–F).

Soft tissues such as muscles are hard to reconstruct while the muscle force has a vital influence on finite element results. Moreover, the characteristics of muscle force are alterability and nonlinear. Here, the way of the follower load was used as an alternative mode to muscle force (Figure 4D). As published by Patwardhan et al. (Patwardhan et al., 2010), using a follower load provided a similar kinematics response as *in vivo*. A thermosensitive truss can transmit force by the principle that expansion with heat and contraction with cold. The thermal expansion effect can transfer the biomechanical load among the elements by assigning a temperature field change to a thermosensitive truss. The expansion coefficient *α* was defined for the prestressed element, and the unbonded prestress was determined using the following formula (Yoganandan et al., 2000):

δT =ε/α (1)

Where δT is the thermal load for the iteration, ε is the thermal strain (growing strains) for the iteration, and *α* is an arbitrary number representing the thermal expansion coefficient.

The follower load of each vertebra is set based on Pasha’s study and is shown in Table 2. Only the T1-S1 vertebral weight was defined in the literature (Pasha et al., 2014) and the percentage of body weight (BW) of the T1 segment was set to (1.1% + 8% of head weight). Thus, the C1-C7 vertebral weight to BW was defined as approximately 1.1%. In other words, the C6 and C7 vertebral weight of BW was set to 6.9 and 1.1%. Additionally, the T3-T5 vertebral weight was larger than T2 and T6 because the weight of superior limbs was taken into account. (El-Rich and Shirazi-Adl, 2005).

**TABLE 2:** The follower load of C6-S1 spinal segments (Pasha et al., 2014).

Vertebral	Percentage of BW (%) in Pasha’s study	Percentage of BW (%) in this study	Follower load(N) (Patient’s BW = 300N)
C6	—	6.9 (Head)	20.7
C7	—	1.1	3.3
T1	1.1 + 8 (Head)	1.1	3.3
T2	1.1		3.3
T3	1.3 + 4.0 (Superior limbs)		15.9
T4	1.3 + 4.0 (Superior limbs)		15.9
T5	1.3 + 4.0 (Superior limbs)		15.9
T6	1.3		3.9
T7	1.4		4.2
T8	1.5		4.5
T9	1.6		4.8
T10	2.0		6.0
T11	2.1		6.3
T12	2.5		7.5
L1	2.4		7.2
L2	2.4		7.2
L3	2.3		6.9
L4	2.6		7.8
L5	2.6		7.8
S1	2.6		7.8

Validation is an important step in building the credibility of numerical models (Babuska and Oden, 2004; Henninger et al., 2010). It is mainly used to verify that the material properties and spinal basic structure are correct. The spinal constraint settings used in validation were shown in Figure 4G. The grid cells of the C6 upper surface were coupled and applied a pure torque (0.5, 1.0, 1.5 and 2.0 Nm) in flexion and extension directions. The lower surface of C7 was bound with the ground. Finally, the angular displacement of the C6-C7 segment was achieved and it displayed that the average error between the experiment and simulation in flexion and extension is 5.7% and 6.9% (Figure 4H) (Li et al., 2019). In addition, as shown in Figure 1, the mean error between the Cobb angles of the reconstructed model and CT images is only 1.08°.

## Result

### Spinal kinematic and biomechanical response after distraction surgery

**FIGURE 5:**
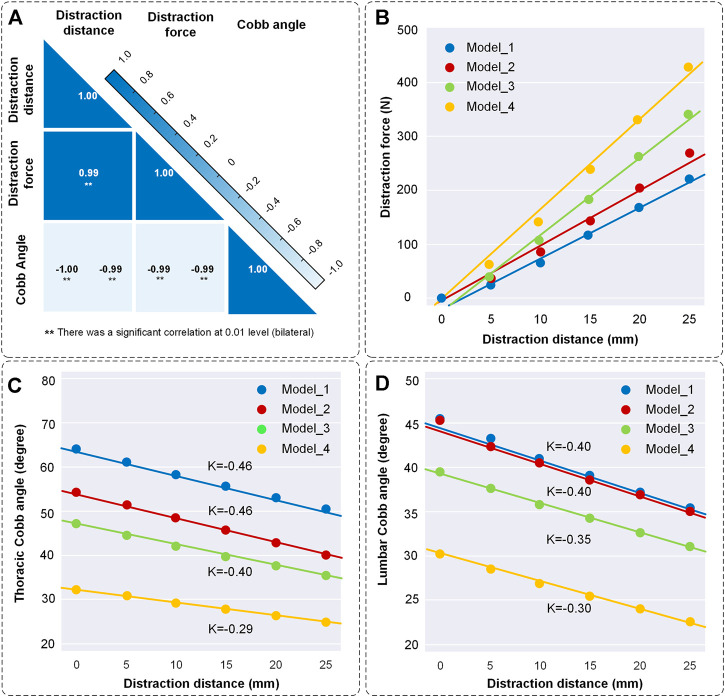
Changes of distraction force and the Cobb angle of thoracic and lumbar curves. **(A)** Correlation coefficient heatmap. The liner relations between distraction distance, distraction force and Cobb angle were normally distributed and significantly correlated at 0.01 level according to the Shapiro-Wilk test and Pearson correlation. **(B)** Scatter diagram of distraction distance and distraction force. K represents the slope of the curves. **(C)** Scatter diagram of distraction distance and the Cobb angle of thoracic scoliosis. **(D)** Scatter diagram of distraction distance and the Cobb angle of lumbar scoliosis. The straight was fitted by scatter points.

Kinematic and biomechanical responses to distraction surgery are shown in Figure 5. In this condition, the distraction distance is significantly correlated with the distraction force and Cobb angle in all four models (Figure 5A). When the distraction distance is 25 mm, the distraction force reaches the maximum (221 N of Model_1, 265 N of Model_2, 343 N of Model_3 and 420 N of Model_4) (Figure 5B). The Cobb angle of thoracic scoliosis and lumbar scoliosis becomes smaller as the distraction distance increases. The slopes of the thoracic curve and lumbar curve are shown (K = −0.46/−0.46/−0.40/−0.29 of the thoracic curve and K = −0.40/−0.40/−0.35/−0.30 of the lumbar curve) (Figures 5C,D).

**FIGURE 6:**
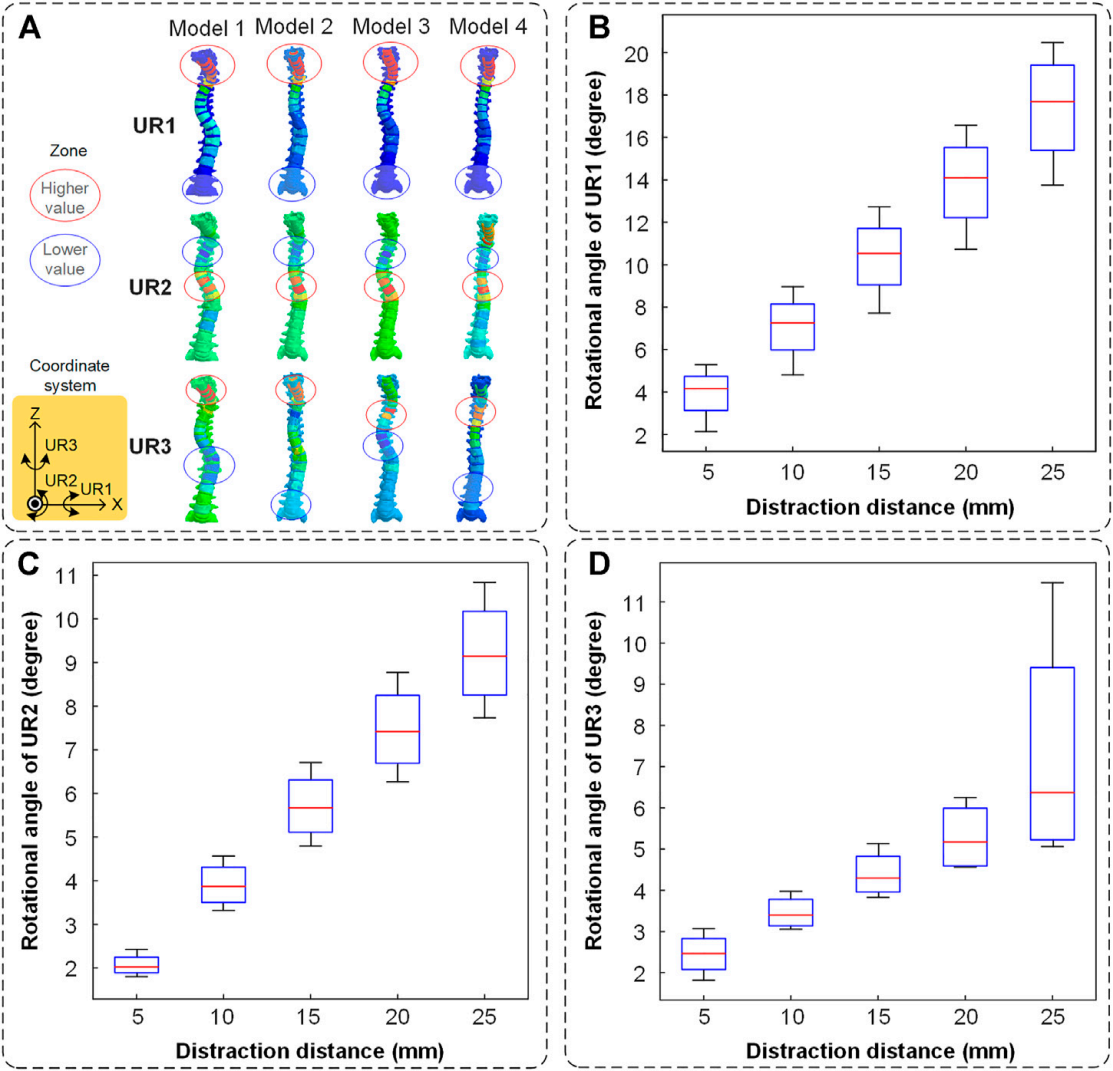
The spinal rotation in three directions. **(A)** The nephogram of finite element results (when the distraction distance is 25 mm). The higher value is labeled in a red circle and the lower value is labeled in a blue circle. **(B)** The spinal rotation in the UR1 direction. **(C)** The spinal rotation in the UR2 direction. **(D)** The spinal rotation in the UR3 direction.

The spinal rotation in three-dimensional directions is shown in Figure 6. The nephogram illustrated that the spinal middle-upper parts had a higher rotational value and the spinal middle-lower parts had a lower one (Figure 6A). In the UR1 direction, the cervical segment had the largest motion range which characterized cervical lordosis. In the UR2 direction, the thoracic segments and lumbar segments had an opposite and obvious movement which displayed a process of straightening the spine. In the UR3 direction, the positions of maximum rotation on the spine were inconsistent. The mean values of the rotational angle increased almost linearly as the distraction distance increased. The maximum values of rotational angle were recorded at each distraction. The rotational angle is the largest (17.68° in UR1, 9.14° in UR2, and 6.36° in UR3) when the distraction distance is 25 mm. The spine had maximum distraction efficiency in the UR1 direction, followed by UR2 and UR3 (Figures 6B–D).

**FIGURE 7:**
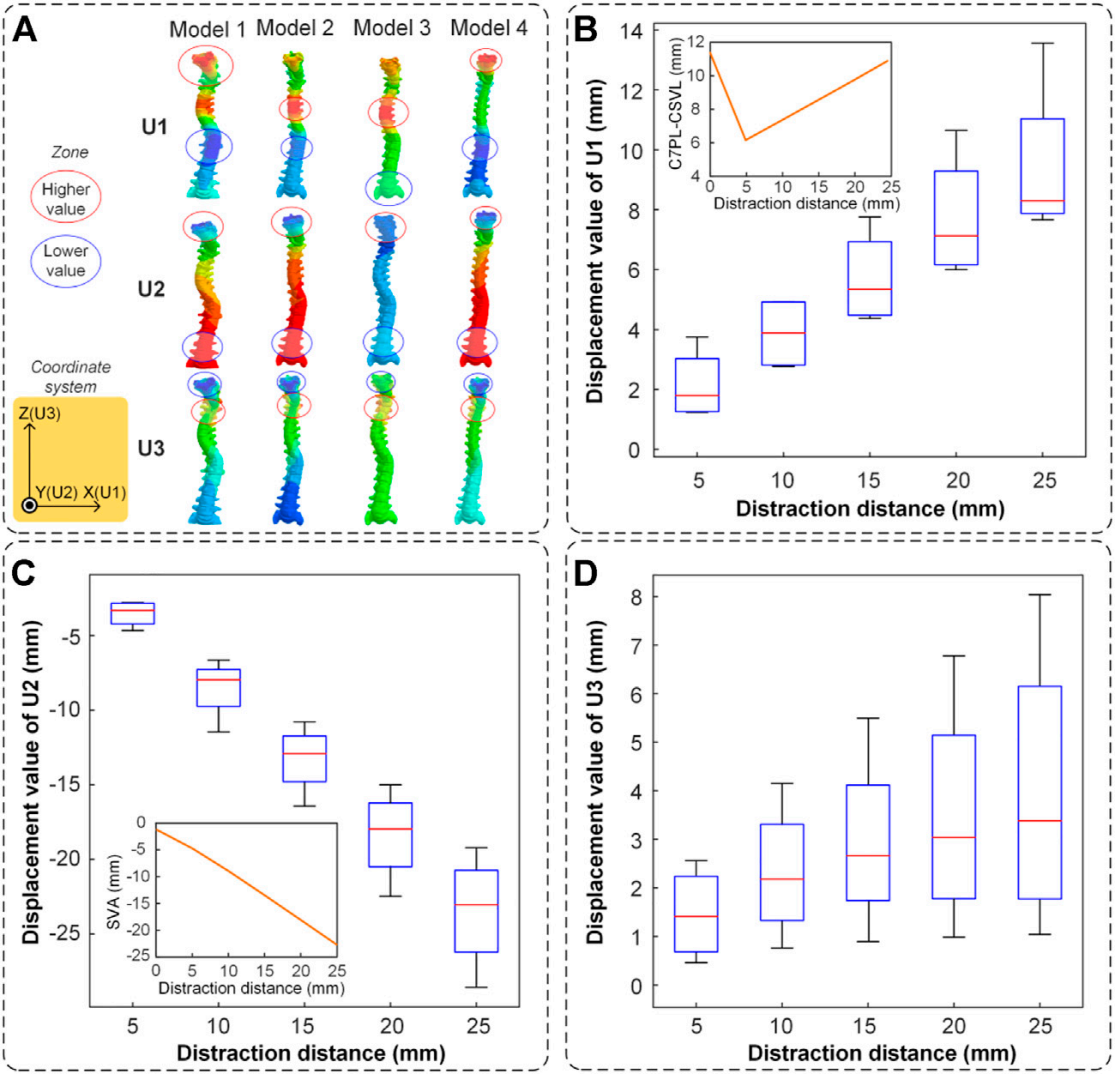
The spinal displacement in three directions. **(A)** The nephogram of finite element results (when the distraction distance is 25 mm). The higher value is labeled in a red circle and the lower value is labeled in a blue circle. **(B)** The spinal maximum displacement in the U1 direction. The mean value of C7PL-CSVL was measured and displayed in the subgraph. **(C)** The spinal maximum displacement in the U2 direction. The mean value of SVA was measured and displayed in the subgraph. **(D)** The spinal maximum displacement in the U3 direction.

The spinal displacement of four models in three directions is shown in Figure 7. The nephogram illustrated that the largest deformation occurred in the middle-upper spine (Figure 7A). In the U1 direction, the cervical and thoracic segments had the largest motion range which characterized the C7PL-CSVL getting greater. In the U2 direction, the cervical segment had the largest negative movement value which displayed the process of cervical lordosis. In the U3 direction, there was an obvious movement in the cervical segment which represented the progress of the spine getting straight.

It is noteworthy that the presence of cervical lordosis caused the downward displacement of the spinal topmost segment. The displacement is significantly related to the distraction distance. The displacement value is the largest when the distraction distance is 25 mm (8.29 mm in U1, −23.05 mm in U2 and 3.38 mm in U3). The maximum distraction efficiency occurred in U2, followed by U1 and U3 (Figures 7B–D). The mean values of C7PL-CSVL and SVA of the four models were shown in the subgraph of Figure 7B and Figure 7C. The coronal balance decreased first and then increased (the minimum value is 6.2 mm when distraction distance is 5 mm) and the sagittal balance (SVA) increased reversely (from −2 mm to −22 mm).

**FIGURE 8:**
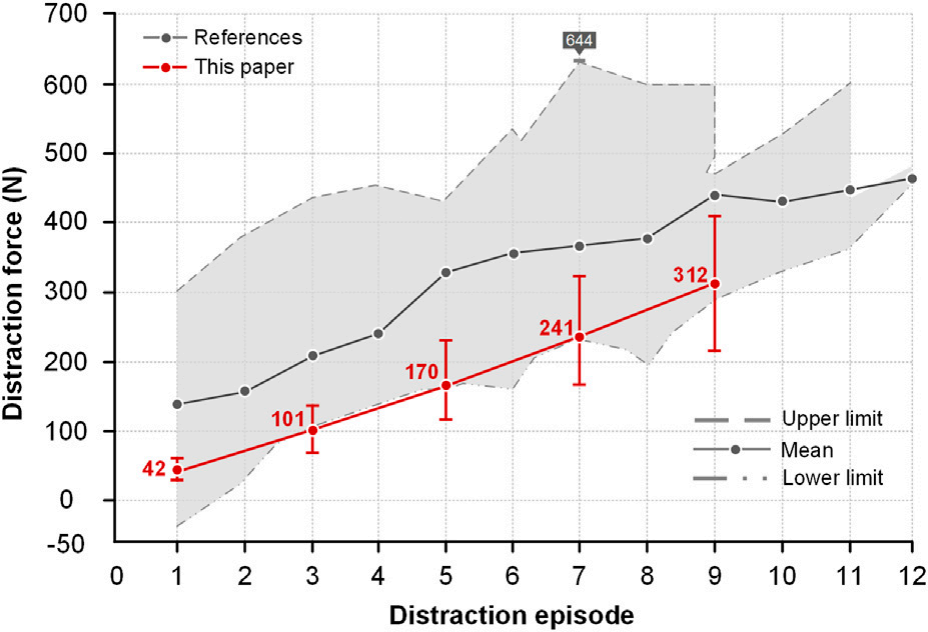
Comparison of distraction force between this paper and literature. The maximum and minimum values were recorded from the literature and were merged into a gray region. The upper boundary of the gray region represented the largest distraction force and the lower boundary represented the smallest one. Accordingly, the gray line represents the mean force value in literature. The upper and lower limits of four models were also recorded and the red line was used to display the mean value in this paper.

Some researchers investigated how the forces increased during every distraction episode (Figure 8). Each distraction force was recorded and the maximum and minimum distraction forces formed the grey shaded area. The maximum distraction force in literature was 644 N (Noordeen et al., 2011) and the one in this paper was 420 N. The trend of mean distraction forces in this paper is consistent with the literature that they increased as the distraction episode increased. The mean distraction forces in this paper are all in the gray shaded area (Noordeen et al., 2011; Teli et al., 2012; Agarwal et al., 2018).

**FIGURE 9:**
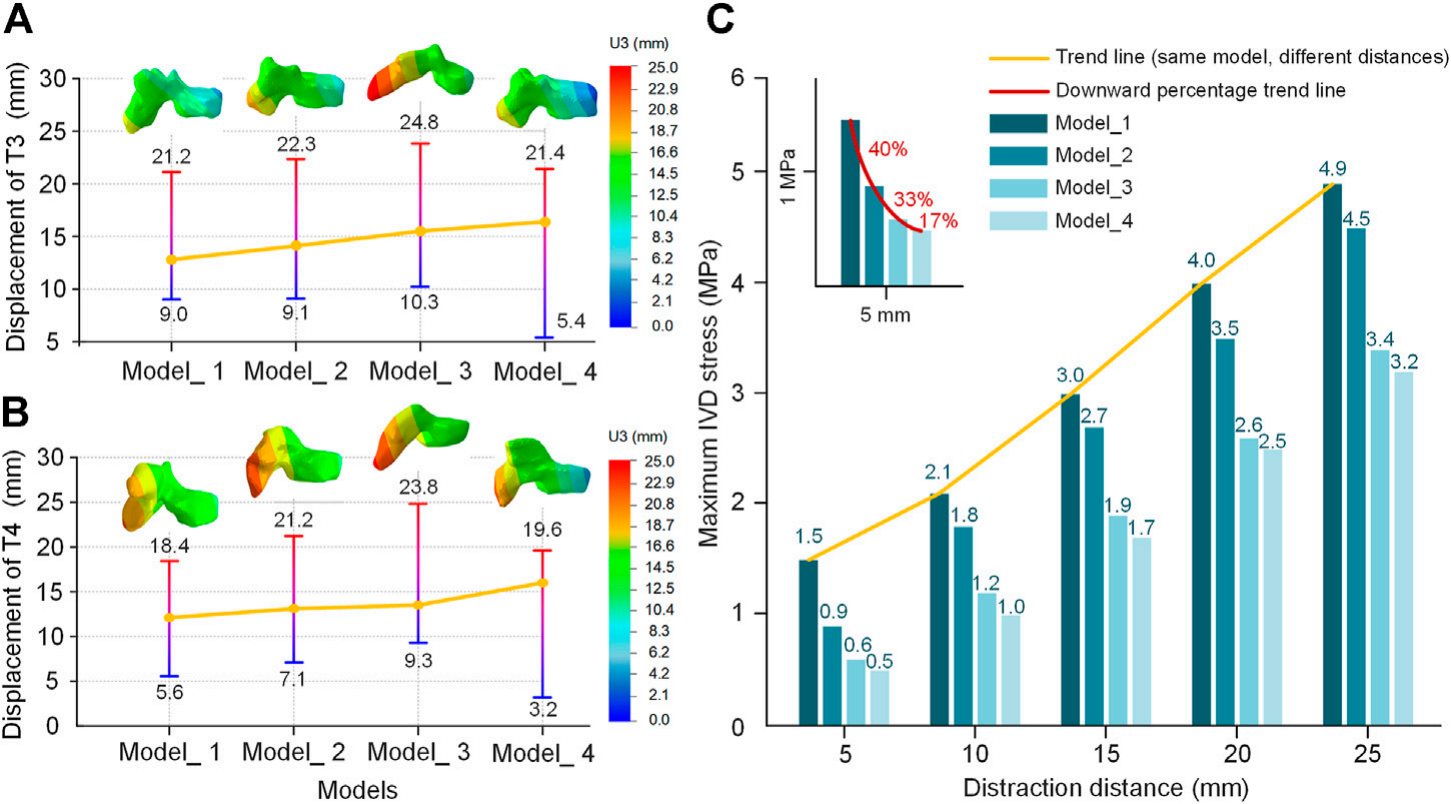
The displacement of the T3 and T4 vertebra in the U3 direction (distraction distance is 25 mm). **(A)** Displacement of T3. **(B)** Displacement of T4. **(C)** The maximum IVD stress of four models from different growth phases at different distraction distances. The asymptotes represent changes going from a low value (blue) to a high value (red). The yellow line is the difference between the low value and high value.

The movement of fixed segments is worth studying to understand the whole spinal movement. Thus, the displacements of T3 and T4 are shown in Figure 9. Significant changes occurred in the anterior and posterior parts of the vertebra. The anterior part had a lower displacement and the posterior part had a larger one. The difference between the highest value and the lowest value represents the middle-part rotation of the vertebra (T3: 12.2/13.2/14.5/16.0 mm in four models; T4: 12.8/14.1/14.5/16.4 mm in four models). The initial Cobb angle is inversely proportional to the difference (Figures 9A,B). The model in late growth phases had the smallest maximum IVD stress at the same distraction distance (Figure 9C). The downward percentage for each period has been shown 40%/33%/17% (5 mm), 14%/33%/17% (10 mm), 10%/30%/11% (15 mm), 13%/26%/3% (20 mm) and 8%/24%/6% (25 mm) (Figure 9C).

**FIGURE 10:**
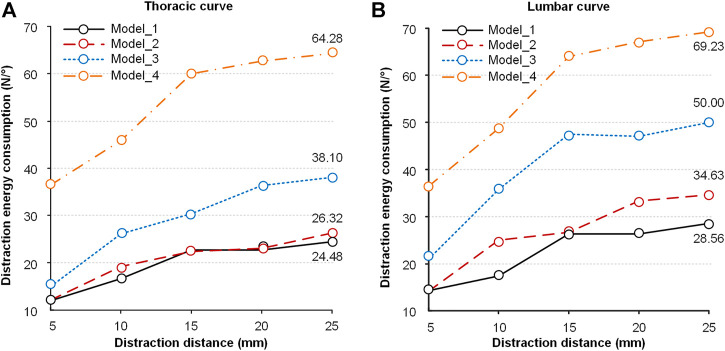
Distraction energy consumption of the four models in the process of distraction. **(A)** The distraction energy consumption in the thoracic curve. **(B)** The distraction energy consumption in the lumbar curve.

Distraction energy consumption is an index that evaluates the distraction force required for each 1° reduction of the Cobb angle. The index of the thoracic curve and lumbar curve is shown in Figure 10. The distraction energy consumption of the four models had the following trend: Model_1 < Model_2 < Model_3 < Model_4 (Figures 10A,B). Every curve had an upward trend, especially with a high slope at the distraction distance of 5–15 mm. The distraction energy consumption was the largest (thoracic curve: 64.28N/° of Model_4, 38.10N/° of Model_3, 26.32N/° of Model_2 and 24.48N/° of Model_1; lumbar curve: 69.23N/° of Model_4, 50.00N/° of Model_3, 34.63N/° of Model_2 and 28.56N/° of Model_1) when the distraction distance was 25 mm.

**FIGURE 11:**
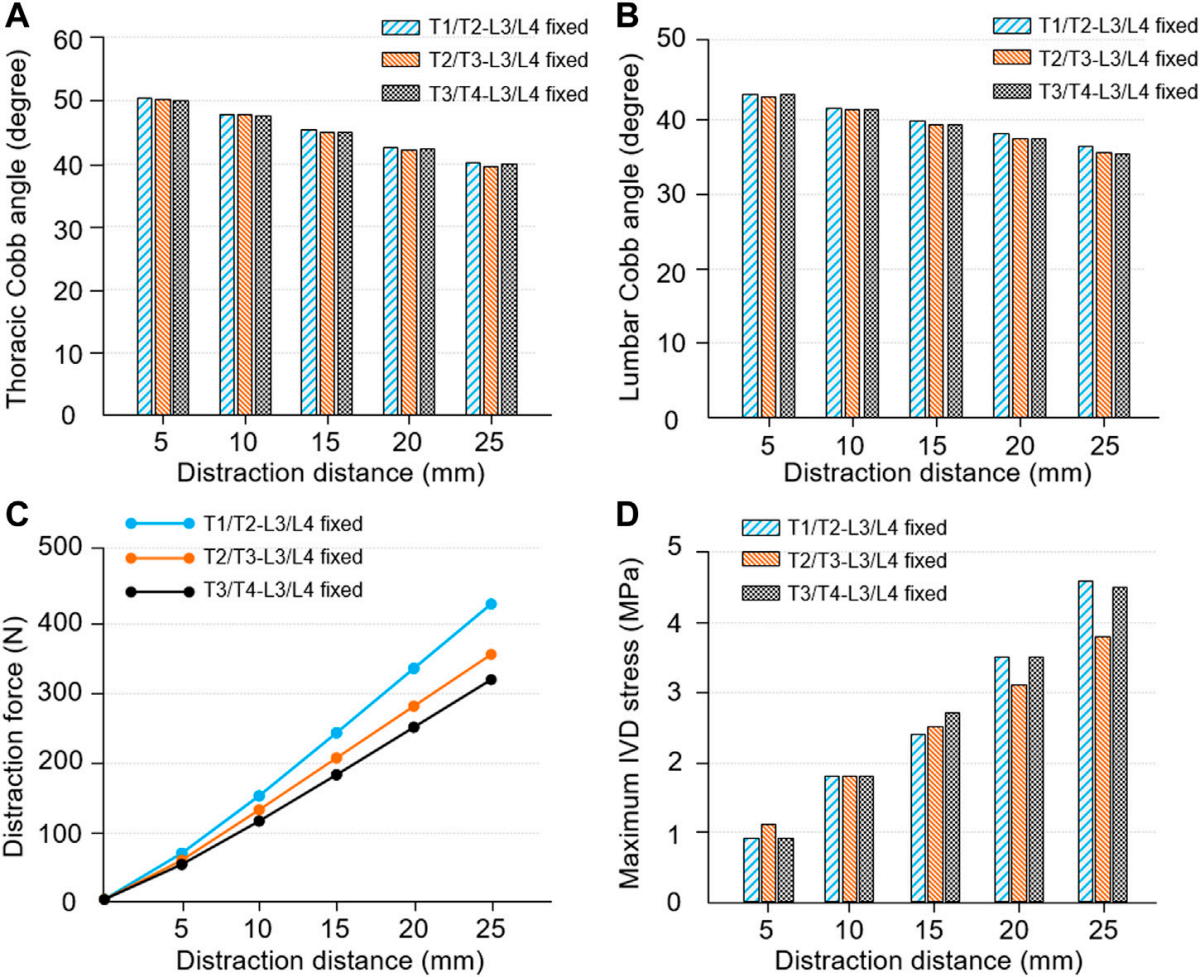
Spinal kinematic and mechanical responses in different fixed modes. **(A)** The changes of thoracic Cobb angle as the distraction distance increased. **(B)** The changes of lumbar Cobb angle as the distraction distance increased. **(C)** The relationship between distraction distance and distraction force. **(D)** The relationship between distraction distance and maximum IVD stress.

Spinal kinematic and biomechanical responses in different fixed modes are shown in Figure 11. The thoracic and lumbar Cobb angles were all decreased as the distraction distance increased. The Cobb angle of the thoracic and lumbar curve was reduced (12.9°/9.4° in T1/T2-L3/L4 fixed-mode group; 13.5°/10.2° in T2/T3-L3/L4 fixed-mode group; 13.2°/10.4° in T3/T4-L3/L4 fixed-mode group) and the correct rate was increased (24.3%/20.5% in T1/T2-L3/L4 fixed-mode group; 25.5%/22.3% in T2/T3-L3/L4 fixed-mode group; 24.8%/22.6% in T3/T4-L3/L4 fixed-mode group) when the growing rod was extended to 25 mm. No significant differences were seen in the groups. The Cobb angle of the T2/T3-L3/L4 fixed-mode group is slightly lower than the other groups (Figures 11A,B). When the distraction distance was less than 5 mm, there was little difference in the distraction force among the three groups which were all less than 65 N. Subsequently, the difference became greater because the slope increased with every 5 mm distraction. The maximum distraction force in the three groups was 311.5, 354.3 and 420.1 N (Figure 11C). The maximum IVD stress is proportional to the distraction distance in the three groups, in which the maximum stress was located in the lower thoracic segment (T5-T10). No obvious pattern was observed in the three groups (Figure 11D).

## Discussion

Periodical and consecutive distraction is an effective treatment for severe EOS, which enables the spinal coronal and sagittal plane deformity correction. The position of each distraction directly determines the rate of rod fracture, which is related to the distraction force. However, more attention has been paid to the growing rod and the spinal biomechanical environment was ignored. Cheung et al. reported complications occur in 60% of patients, including distraction failure, proximal junctional kyphosis (PJK) and implant loosening. These patients all needed reoperation via the extension of the proximal foundation or exchanging larger screws (Cheung et al., 2019). Watanabe et al. performed a retrospective multicenter review of 88 patients with EOS and found that 23% of patients developed PJK (Watanabe et al., 2016). The occurrence of PJK is due to the ossification in fixed segments and motor compensation in unfixed segments. These phenomena cause reoperation that is related to surgical phases and distraction mode. Thus, more attention paid to the kinematic and biomechanical environment in local structure is momentous for understanding complications unrelated to the rod itself.

Many researchers have tried to judge the optimal distraction force by whether the rod is broken or not (Teli et al., 2012; Agarwal et al., 2017a). They ignored whether the spinal coronal and sagittal plane was still balanced. Other researchers also focused on the effect of distraction frequency on the reduction of rod fracture. Agarwal et al.(2015) confirmed the importance of a shorter distraction period in reducing stresses on the rods. But a shorter distraction frequency resulted in multiple operations, which lead to greater injury for patients. Magnetically driven growing rods as an alternative to traditional growing rod technology can increase the distraction frequency and reduce surgical injury at the same time (Agarwal et al., 2014a). It is also not popular because of its limited distraction force and high rod breaking rate (Rushton et al., 2019). Fortunately, Agarwal et al. (2015) mentioned a great sagittal balance of the spine in optimal distraction force and the reduction of Cobb angle. But they have not revealed the law of diminishing returns that the reduction rate of Cobb angle decreased with the increase of distraction times (Sankar et al., 2011). The present study aimed to investigate the kinematic and biomechanical response of the spine after traditional growing rod surgery. To achieve this, distraction force, spinal displacement and rotation in three-dimensional directions, reduction of thoracic and lumbar Cobb angle and distraction energy consumption were measured.

As an initial factor, distraction distance affected the force on the growing rod and the spinal shape. Model_4 had the minimum initial Cobb angle and the largest distraction force. Understandably, the downward component of the muscle force on the vertebra increases with the decrease of Cobb angle, which made the upward support force smaller (Figure 5B). Some studies had shown that large displacement was achieved with a relatively small distraction force (Noordeen et al., 2011; Shekouhi et al., 2022). This condition commonly occurred in the prophase distraction. However, there is a lack of sufficient statistical data to establish a clear timeline or a threshold of distraction distance. There are various variables that could affect the conclusion, including the initial Cobb angles, number of fixed segments, time of the first implantation and distraction frequency, etc. Although lacking sufficient evidence, it can be considered that a small distraction force is accompanied by a large displacement when the distraction distance is 0–15 mm. Additionally, the spinal stiffness was increased due to the spine growing itself and skeletal maturity, which caused a greater distraction force (Noordeen et al., 2011). The reduction trend of thoracic and lumbar Cobb angle was consistent that Model_4 had the minimal Cobb angle changes. The trend confirmed the law of diminishing returns (Figures 5C,D). These results enlightened us that a reduced distraction distance should be appropriately performed in spine straightening gradually. At the same time, the time interval of distraction should be prolonged to 6–9 months in every distraction.

Positive benefits of spinal distraction should be the rotation of each vertebra in the UR2 direction. Conversely, spinal rotation in the UR1 direction represented cervical lordoses such as flexion or extension, and the one in the UR3 direction represented the left-right torsion of the human body (Figure 6A). The rotational situation exhibited that cervical lordosis had the largest change in the distraction process and self-rotation inevitably occurred. The negative benefits of rotation basically occurred in the cervical spine, especially the segment unfixed. The thoracic vertebra and lumbar vertebra had an opposite rotational direction and that is why the thoracic and lumbar scoliosis is decreasing (Figure 6). The positive benefits of spinal distraction should be the distance of each vertebra in the U3 direction. Additionally, the spinal movement in the U1 direction represents a horizontal displacement of the cervical thoracic segment in the coronal plane. The coronal balance parameter C7PL-CSVL decreased first and increased (Figures 7A,B). The spinal movement in the U2 direction represents the trend of cervical flexion. The sagittal balance parameter SVA became greater (Figures 7A,C). It reminds us that a single distraction should not be too large, otherwise the coronal and sagittal balance will backfire.

In the movement of the U3 direction, the thoracic segment had a forward displacement and the cervical segment had a reverse displacement. It explained that spinal scoliosis was gradually improving, while the existence of the original cervical curvature caused it to move forward and downward. This phenomenon is consistent with the rotational situation. Another reason for this phenomenon is that the distraction force acted on the posterior vertebra rather than the vertebra center, which caused an additional displacement of the posterior vertebra. The U3 displacement of the T3-T4 anterior and posterior vertebra showed an upward trend and reached the maximum in Model_4 (Figure 9). For the other models, Model_4 has less displaceable space in the +U3 direction and a more displaceable space in the −U3 direction. The maximum IVD stress also has been measured, which was an index that characterized the rate of complications to some extent. The trend showed that later implantation of the growing rod can effectively reduce the maximum IVD stress, or reduce the rate of complication (Figure 9C). Bess et al. (2010) demonstrated that rates could be reduced by delaying initial implantation (13% decrease for every additional year of inpatient age at the beginning of treatment).

Distraction energy consumption is very important for understanding the law of diminishing returns (Figure 10). It represents the distraction force needed for each 1° distraction. For all models, the distraction energy of Model_4 is the largest, followed by Model_3, Model_2, and Model_1. This law enlightens us that an optimal distraction distance is accompanied by a lower distraction force and a better correction effect, rather than the largest distraction force. This is why some researchers believe applying less distraction with more frequent surgeries is favorable. However, additional surgeries would increase the risk of complications such as wound infection (Bess et al., 2010; Agarwal et al., 2015, 2019). This risk increased by 24% at each additional surgery, which had been shown by authors (Bess et al., 2010; Mundis et al., 2013).

The influences of fixed segments on the kinematic and biomechanical response have been studied (Figure 11). It exhibited that the intragroup trend was consistent with the trend mentioned above, while the difference between groups is not obvious. Among the results, the curve of distraction force had a gradually rising slope and the T3/T4-L3/L4 fixed model had the largest distraction force (Pei et al., 2022). The spinal structure itself has a stage of easy deformation due to the elasticity of soft tissue, which includes stages of compression and tension. Thus, the more the vertebral numbers in the fixed area, the larger the deformable space.

After understanding the law of diminishing returns, there is some specific advice for surgeons. Although the time interval of distraction has been proposed to be 6 months in early experience with growing rod procedures, more and more surgeons are used to extending the frequency to 9–12 months/time. Because continued forceful distraction in dysplastic spine theoretically includes PJK. More recently, other authors have reported similar experiences. Carbone et al. (2019) reported a 1-year distraction interval in their cohort of NF1 patients. They explained that less frequent lengthening surgeries reduce the psychological burden on their patient. Another reason for the long distraction interval used in this cohort was that the risk of wound infection is minimized by reducing the frequency of distraction. Additionally, a single over-distraction will backfire especially in spine straightening. Thus, decreasing distraction distance and increasing interval may be considered by the surgeons to reduce the rod fracture and complications.

There are a few limitations to this study. First, the distraction amounts of bilateral growing rods were not adjusted according to the initial state of the model, which should be considered during the operation, resulting in additional imbalance. Second, there were no corresponding biomechanical experiments to verify the finite element results. It was very difficult to obtain a long segmental specimen of scoliosis in children. Third, material properties that change with age were not considered. Finally, this paper did not consider other types of scoliosis, which may lead to accidental results. While the distraction distance and distraction frequency are vital to understanding rod fracture, there are other factors that need further investigation such as the patient’s age (Bess et al., 2010; Jiang et al., 2011; Upasani et al., 2016) and T1-S1 growth rate (Abolaeha et al., 2012; Agarwal, 2015, 2015).

## Conclusion

The kinematic and biomechanical responses of the spine occurring after growing rod distraction surgery were investigated. Compared to previous studies, a more realistic spinal environment was restored, being simulated the postoperative effects of different growth phases and different fixed segments on spinal distraction. Our results show that the process of spinal distraction may be accompanied by the spinal re-imbalance in the coronal sagittal plane. The positive distraction benefits of the spine are inversely proportional to the distraction distance. In addition, there is an optimal distraction force, rather than the maximum one, to ensure lower distraction energy consumption and lower pressure on the rod and IVD. The choice of optimal distraction force depends on the response of the fixed segment and the positive return we obtained. In summary, more attention should be paid to the spinal balance and aesthetic evaluation, rather than relying on whether the rod is broken to set the optimal distraction force. This study can provide a better understanding of the biomechanical response after spinal distraction surgery. The next avenue of future work could be adding more types of the spine to verify our results.

## Data Availability

The raw data supporting the conclusions of this article will be made available by the authors, without undue reservation.
